# SARS-CoV-2 infection-induced immunity reduces rates of reinfection and hospitalization caused by the Delta or Omicron variants

**DOI:** 10.1080/22221751.2023.2169198

**Published:** 2023-03-01

**Authors:** Marc-Antoine de La Vega, Efstathia Polychronopoulou, Ara XIII, Zhe Ding, Tong Chen, Qixing Liu, Jiaming Lan, Marie-Edith Nepveu-Traversy, Hugues Fausther-Bovendo, Mohammed F. Zaidan, Gary Wong, Gulshan Sharma, Gary P. Kobinger

**Affiliations:** a Galveston National Laboratory, Department of Microbiology and Immunology, Institute for Human Infections and Immunity, University of Texas Medical Branch, Galveston, TX, USA; b Office of Biostatistics, University of Texas Medical Branch, Galveston, TX, USA; c Viral Hemorrhagic Fevers Research Unit, CAS Key Laboratory of Molecular Virology and Immunology, Institut Pasteur of Shanghai, Chinese Academy of Sciences, Shanghai, People’s Republic of China; d University of Chinese Academy of Sciences, Beijing, People’s Republic of China; e Global Urgent and Advanced Research and Development (GuardRX), Batiscan, Canada; f Department of Internal Medicine, Division of Pulmonary, Critical Care, & Sleep Medicine, University of Texas Medical Branch, Galveston, TX, USA; g Department of Internal Medicine, University of Texas Medical Branch, Galveston, TX, USA

**Keywords:** COVID-19, vaccination, infection-induced immunity, vaccine-induced immunity, hybrid immunity

## Abstract

During a pandemic, effective vaccines are typically in short supply, particularly at onset intervals when the wave is accelerating. We conducted an observational, retrospective analysis of aggregated data from all patients who tested positive for SARS-CoV-2 during the waves caused by the Delta and Omicron variants, stratified based on their known previous infection and vaccination status, throughout the University of Texas Medical Branch (UTMB) network. Next, the immunity statuses within each medical parameter were compared to naïve individuals for the effective decrease of occurrence. Lastly, we conducted studies using mice and pre-pandemic human samples for IgG responses to viral nucleocapsid compared to spike protein toward showing a functional component supportive of the medical data results in relation to the immunity types. During the Delta and Omicron waves, both infection-induced and hybrid immunities were associated with a trend of equal or greater decrease of occurrence than vaccine-induced immunity in hospitalizations, intensive care unit admissions, and deaths in comparison to those without pre-existing immunity, with hybrid immunity often trending with the greatest decrease. Compared to individuals without pre-existing immunity, those vaccinated against SARS-CoV-2 had a significantly reduced incidence of COVID-19, as well as all subsequent medical parameters. Though vaccination best reduces health risks associated with initial infection toward acquiring immunity, our findings suggest infection-induced immunity is as or more effective than vaccination in reducing the severity of reinfection from the Delta or Omicron variants, which should inform public health response at pandemic onset, particularly when triaging towards the allotment of in-demand vaccinations.

## Introduction

In 2020, the COVID-19 pandemic caused by SARS-CoV-2 spread worldwide, yet by 2021 expectations remained high that the first doses of vaccines would soon be deployed on a global scale. While the safety and immunogenicity of these vaccines had been evaluated in clinical trials, their ultimate contribution to combating the pandemic remained uncertain. Indeed, data from previous studies hinted at a short-lived coronavirus immunity. For example, a previous challenge experiment with HCoV-229E in humans displayed that at 12 months following the initial infection, rechallenge of individuals exhibiting antibody levels marginally above the baseline with the same virus resulted in reinfection, although the subjects exhibited a reduced shedding period and none became clinically ill [1]. Furthermore, the protective immunity against natural infection with seasonal coronaviruses (HCoV-NL63, −229E, -OC43, and -HKU1) has been shown to be similar, where reinfection with the homologous virus was frequently observed after a 12-month period in individuals followed over 35 years [2]. Likewise, neutralizing antibody (NAb) levels in individuals recovering from a low peak-infective dose of SARS-CoV-2 were found to decline to titres approaching baseline levels within three months [3].

Conversely, individuals with laboratory-confirmed SARS-CoV-2 and requiring hospitalization maintained higher levels of antibodies throughout the same three-month period, with one study showing levels to persist for at least 13 months [4]. Interestingly, following even a mild infection with SARS-CoV-2, long-lived bone marrow plasma cells have been detected at 11 months post-infection [5]. Notably, studies regarding other pandemic-prone coronaviruses, such as SARS-CoV and MERS-CoV, have shown that specific IgG and NAbs could be detected in subjects infected two to three years prior, before the levels decline [6,7]. Furthermore, a recent study demonstrated that individuals who had previously been infected with SARS-CoV and immunized during this pandemic with the BNT162b2 messenger RNA (mRNA) vaccine against SARS-CoV-2, developed potent cross-clade pan-sarbecovirus NAbs [8]. These antibodies were found to be capable of neutralizing not only numerous SARS-CoV-2 variants of concerns (VOCs), but also bat- and pangolin-associated coronaviruses. This notion of pre-exposure immunity from past infections was recently reinforced in the context of SARS-CoV-2, in which data from California and New York between May to November 2021 showed that infection-induced, vaccine-induced, and hybrid immunity all better protected against COVID-19 compared to those previously unexposed to SARS-CoV-2, with data from the week of May 30 showing infection-induced immunity conferred comparable protection to hybrid immunity [9].

The constant emergence of novel VOCs has now become a hallmark of this prolonged pandemic and continues to drive the infection waves. These variants ultimately become dominant across the globe before being outcompeted by a subsequent VOC. The impact of a variant on public health can be quantified in relation to their association with hospitalizations, intensive care unit (ICU) admissions, and deaths, providing a benchmark to assess COVID-19 severity. Alternatively, factors leading to emergency department visits mitigate the use of this parameter towards quantifying severity, as hospitalizations and intensive care unit admission are initiated after professional assessment, whereas emergency department visits lack such controls. For example, vaccinated individuals may be more prone to seek healthcare even for minor symptoms compared to the unvaccinated; or conversely, the unvaccinated may be worried during local upticks of infection compared to vaccinated individuals. Many factors confound the use of emergency department visits as a control for disease severity, but its conversion into hospitalizations, ICU admissions, and deaths is robust. Hence, an analysis leading to optimized vaccine allocation should weight more heavily the latter three parameters.

While the efficacy of vaccination and boosters clearly have demonstrated their impact at reducing the incidence and severity of disease, healthcare policies can benefit from immunity data to inform optimal vaccine allocation, particularly during pandemic onset when vaccines are scarce. Here, we performed a retrospective analysis of SARS-CoV-2 laboratory-confirmed infections, along with number of infections, hospitalizations, ICU admissions, and deaths among patients of the UTMB network during the Delta and Omicron waves. Clinical outcomes were analysed in relation to individuals stratified in groups: no prior immunity, vaccine-induced, infection-induced, and hybrid immunity. Lastly, we present mouse and human data describing the potential of immunity against the nucleocapsid of SARS-CoV-2 in support of the observations associated with increased protection of infection-induced and hybrid immunity.

## Materials and methods

### Study design

We included all patients who were diagnosed with COVID-19 at any UTMB facility throughout the entire Southeast Texas network, between 1 July 2021, and 31 October 2021, as well as between 10 December 2021, and 28 February 2022, coinciding with the two prominent waves of VOC. The catchment area of UTMB includes 17 counties in Southeast Texas and encompasses approximately 1 790 008 individuals [10]. COVID-19 diagnosis required a positive molecular (Abbot RealTime SARS-CoV-2 [Abbott], ID NOW™ COVID-19 [Abbott], Panther Fusion® SARS-CoV-2 Assay [Hologic], Aptima® SARS-CoV-2 assay [Hologic], Xpert® Xpress CoV-2 plus [Cepheid], Xpert® Xpress CoV-2/Flu/RSV plus [Cepheid]) or antigen (BinaxNOW™ COVID-19 antigen self-test [Abbott], SARS-CoV-2 Antigen test [VITROS Immunodiagnostic]) test. Prior infection with COVID-19 was defined as having a prior positive test or a diagnosis of COVID-19 infection in the medical history of the patient both at least 30 days before recorded infections during the waves of interest. Any patient’s further positive COVID-19 test within the 30 days prior to July 1 or December 10 were excluded to maintain the one-to-one relationship of patient infection data to the medical parameters. Both emergency department visits and hospitalizations were considered COVID-19-related if it was the primary diagnosis and occurred within 15 days from a positive test. If a patient had multiple hospital admissions related to COVID-19 symptoms during that period, only the first visit was included. Patients were classified as vaccinated if they had received the recommended doses based on vaccine type and at least 14 days had elapsed from the last dose until COVID-19 infection. ICU patients were a subset of those hospitalized with COVID-19 as the primary diagnosis to remove confounds of including patients admitted to ICU due to another diagnosis, though with COVID-19 that may not have induced an ICU admission, thus not representing an appropriate COVID-19 severity level. COVID-19-associated inpatient deaths were counted exclusively from hospitalizations within the UTMB system and thus excluded deaths at home, in hospices, or in external hospitals. In figures and tables, we define the various immunity types as individuals with no known induced immunity prior to the current infection from vaccination or infection (NoI), immunity induced by vaccination prior to first infection (VaxI); immunity by having recovered from a known previous infection prior to reinfection (InfI); immunity from both vaccination and a known previous infection prior to reinfection (HybrI).

### Regional data

Data on vaccination rates for each of the 17 counties included in the catchment area covered by UTMB comes from a publicly available database (COVID-19 Vaccinations in the United States, County), which is maintained by the Centers for Disease Control and Prevention [11]. The variables considered for analysis were “Series_Complete_Yes,” which represents the total number of individuals who have received, at the minimum, both doses of a two-dose vaccine or a single-dose vaccine, and “Census2019,” which reports the total population of the county based on the 2019 census conducted by the U.S. Census Bureau. These data were subsequently used to calculate the vaccination rate of each county (17) within the UTMB catchment area. From the 17 counties an average vaccination rate was derived and used to calculate a representation of total vaccinated and unvaccinated populations within the UTMB catchment area. These populations were used to calculate the cumulative incidence of cases within the UTMB cohort in vaccinated and unvaccinated individuals. Data regarding cumulative incidence in Texas comes from a publicly available database (csse_covid_19_daily_reports_us), which is maintained by the Johns Hopkins University of Medicine in the context of its Coronavirus Resource Center [12].

### Ethics statement

Ethical approval to collect, use, and publish human data was obtained from the Institutional Review Board at UTMB, Galveston, under the protocol #20-0249, titled “Qualitative Assessment of Individuals with COVID-19 positive test at UTMB.” Data were de-identified prior to analysis and aggregated to maintain privacy. The animal experiments and sample collection were approved by the animal ethics committee at the Pasteur Institute of Shanghai, under the authorization number A2021009.

### Animal experiments

Wild-type, 8-month-old BALB/c mice were purchased from Beijing Vital River Laboratory Animal Technology Co., Ltd. The mice were transduced intranasally with 10^11^ viral particles of Ad5-hACE2 in 50 μl PBS. At day 5 post-transduction, the mice were divided into two groups, the infected group (*n* = 6) and control group (*n* = 5). The infected group were infected intranasally with 10^5^ PFU of HCoV-NL63 in 20 or 50 μl PBS, and the control group with an equal volume of PBS. At day 21 or 35 post-infection, the previous infection procedure was repeated for the second challenge. At day 49 following the second challenge with HCoV-NL63, all mice were sacrificed and the serum was collected to perform the ELISA.

### ELISA

96-well microplates were coated with recombinant nucleocapsid (N) or spike (S) protein (50 ng/well) from HCoV-NL63, HCoV-229E, and SARS-CoV-2, and incubated at 4°C overnight. The plates were washed and blocked with 5% skim milk. The mice serum samples were diluted in triplicate starting at 1:50 with 2-fold serial dilution, before incubation with the coated plates at 37°C for 2 h. After washing four times, the plates were incubated with a 1:30 000 dilution of HRP-conjugated goat anti-mouse IgG (Abcam, Cat. ab6789) at 37°C for 1 h. After washing five times, the plates were visualized with TMB solution (ThermoFisher, Cat. 002023) and stopped with stop solution (Solarbio, Cat. C1058). The absorbance at a wavelength of 450 nm was detected with a microplate reader (Biotek Synergy H1).

IgG levels in human samples from Turkey were evaluated using 96-well plates coated with SARS-CoV-2 N or S recombinant proteins (200 ng/well). The plates were first blocked with PBS-T-M 3% for 2 h at RT, then human serum samples dilutions (1:100 in PBS) were added to wells in duplicate. After an incubation of 2 h at 37°C, plates were washed four times with PBS-T and incubated with HRP-conjugated goat anti-human IgG (1:10 000, Invitrogen, Cat. 31412) for 1 h at 37°C. Plates were washed four times with PBS-T, and TMB (ThermoFisher, Cat. 002023) solution was added and then stopped with HCl 2N. The absorbance (450 nm) was measured using a microplate reader (MBIZLQ100 evolution microplate reader).

### Statistical analysis

Patients were assembled into four groups based on vaccination and prior-infection status. Data are presented for each group in relation to parameters (number of infections, emergency department visits, hospitalizations, ICU admissions, deaths) per Delta or Omicron infection as frequencies and percentages, or frequencies and incidence per 100 000 persons. Chi-squared tests were used to determine statistical significance comparisons between cross-reactivity to the spike and nucleocapsid, as well as to the various immunity types and where every combination of two different groups were compared, as they were presumed random and had fit a 1:1 distribution, from which the *P* value was calculated. Statistical significance between ELISA endpoint titres was determined by two-tailed t-tests. (*p ≤ 0.05, **p ≤ 0.01 ***p ≤ 0.001, ****p ≤ 0.0001).

### Role of the funding source

The funders had no role in the design of the study or the decision to publish this work.

## Results

### Vaccination status of the study population

Overall, 22 407 confirmed cases of COVID-19 were identified for the period spanning 1 July to 31 October 2021, when the Delta VOC was predominant. Of these, 2 543 (11.3%) were fully vaccinated, while 19 864 (88.7%) were unvaccinated, regardless of their status of a previous infection by SARS-CoV-2. As a reference, 53.4% of individuals in the catchment area of UTMB were vaccinated as of 31 October 2021. Among all COVID-19 patients from the retrospective cohort identified for Delta, 642 (2.9%) were unvaccinated but had a known prior infection, whereas 157 (0.7%) vaccinated patients also had a known prior infection (Supplementary Table 1). The cumulative incidence of COVID-19 cases, based on the catchment area of UTMB and expressed per 100 000 individuals, was 266 and 2 383 for vaccinated and unvaccinated individuals, respectively, compared to an overall incidence of 14 617.4 per 100 000 individuals for the same period in Texas.

The period of 10 December 2021, to 28 February 2022, was marked by the dominance of the Omicron VOC and 29 070 confirmed cases were identified, in which 9 349 (32.2%) and 19 721 (67.8%) were vaccinated and unvaccinated, respectively. As of 28 February 2022, the vaccination rate for the catchment area covered by UTMB had increased to 59.5%. Of the 29 070 confirmed cases during that period, 2 222 (7.6%) individuals had infection-induced immunity, while 1 088 (3.7%) exhibited hybrid immunity (Supplementary Table 2). The cumulative incidence expressed per 100 000 individuals for positive cases within the UTMB network increased to 878 and 2 719 for vaccinated and unvaccinated individuals, respectively, while the incidence in Texas was 22 820.9 per 100 000 for the same period.

### Clinical outcomes

During the Delta wave, a total of 938 individuals were hospitalized within the UTMB network due to COVID-19. Of these, 879 (93.7%) had no pre-existing immunity, while vaccine-induced, infection-induced, and hybrid immunities accounted for 51 (5.4%), 7 (0.8%), and 1 (0.1%) hospitalizations, respectively. In relation to ICU, 173 were reported during Delta, whereby individuals with no pre-existing immunity, vaccine-induced, infection-induced, and hybrid immunity accounted for 161 (93.1%), 12 (6.9%), 0, and 0, while the associated number of deaths were 125 (92.6%), 8 (5.9%), 2 (1.5%), and 0, respectively (Supplementary Table 1).

For Omicron, the number of hospitalizations followed a similar trend as Delta based on group stratification whereby of the 451 hospitalizations, 354 were from individuals with no pre-existing immunity (78.5%), 72 with vaccine-induced immunity (16.0%), 18 with infection-induced immunity (4.0%), and 7 with hybrid immunity (1.5%). Similarly, the number of ICU admissions following the same stratification were 61 (88.4%), 6 (8.7%), 2 (2.3%), and 0, accounting for a total of 69, while the number of deaths were 36 (85.7%), 5 (11.9%), 1 (2.4%), and 0, respectively (Supplementary Table 2).

### Infection-induced and hybrid immunities lead to comparable protection against Delta and Omicron

**Figure 1.**
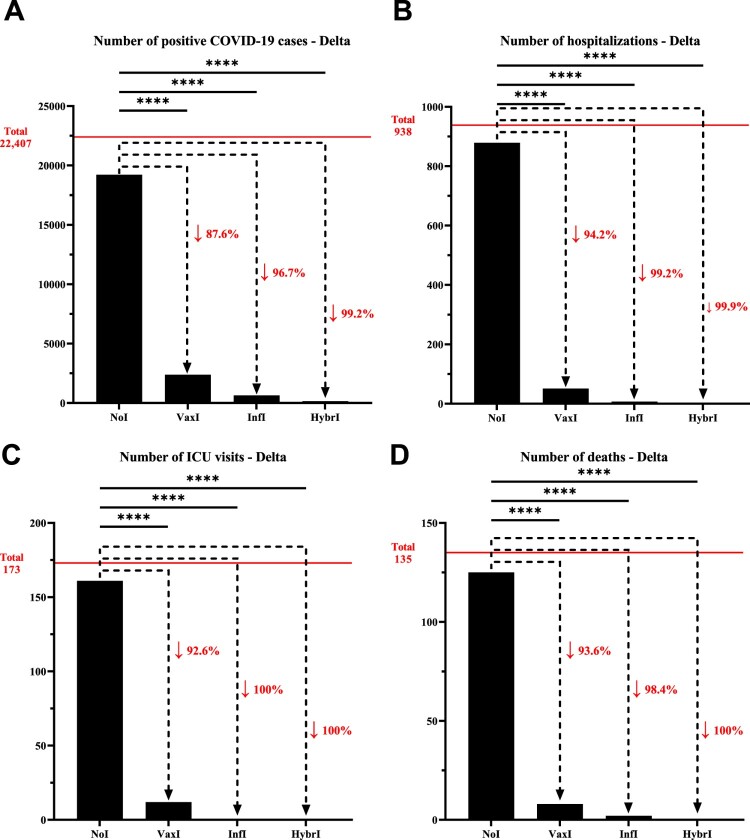
Number of (A) positive COVID-19 cases, (B) hospitalizations, (C) intensive care unit visits, and (D) deaths that have been recorded within the University of Texas Medical Branch network during July 01, 2021, and October 31, 2021, characterized by the predominance of the Delta variant. The horizontal line represents the total number of individuals for each category and the dotted arrows represent the percentage decrease compared to the NoI group. **NoI**: No known induced immunity from reportedly naïve patients who have not been vaccinated and have not experienced a COVID-19 infection prior to the current infection; **VaxI:** Vaccine-induced immunity prior to first infection; **InfI:** Infection-acquired immunity without vaccination but having recovered from a known previous infection prior to reinfection; **HybrI:** Hybrid immunity from both vaccination and a known previous infection prior to reinfection.

To investigate the role of the various immunity types (Vaccine-induced, infection-induced and hybrid), the number of infections, hospitalizations, ICU admissions, and deaths were evaluated during both the Delta and Omicron waves and compared to the group with no pre-existing immunity. In the context of Delta, the number of infections decreased by 87.6% for individuals with vaccine-induced immunity compared to those with no pre-existing immunity, which was more pronounced for infection-induced (96.7%) and hybrid immunity (99.2%). A similar trend was observed for hospitalizations, where a reduction from individuals with no pre-existing immunity of 94.2%, 99.2%, and 99.9% was observed for vaccine-induced, infection-induced, and hybrid immunity, respectively; a pattern that was repeated with respect to ICU admissions and deaths, although within a smaller sample size (Figure 1, Supplementary Table 3).

**Figure 2.**
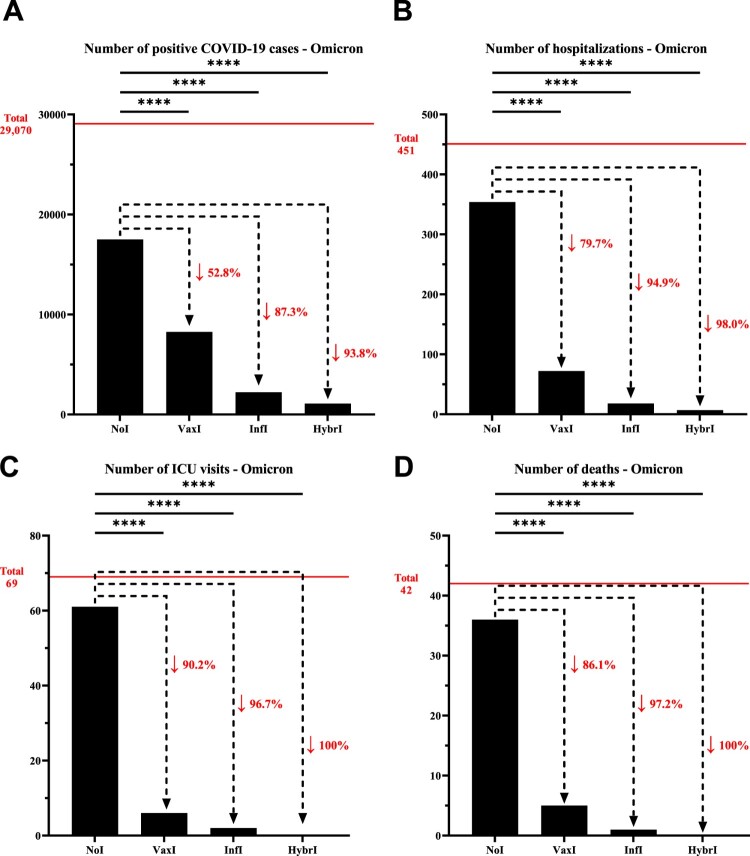
Number of (A) positive COVID-19 cases, (B) hospitalizations, (C) intensive care unit visits, and (D) deaths that have been recorded within the University of Texas Medical Branch network during December 10, 2021, and February 28, 2022, characterized by the predominance of the Omicron variant. The horizontal line represents the total number of individuals for each category and the dotted arrows represent the percentage decrease compared to the NoI group. **NoI**: No known induced immunity from reportedly naïve patients who have not been vaccinated and have not experienced a COVID-19 infection prior to the current infection; **VaxI:** Vaccine-induced immunity prior to first infection; **InfI:** Infection-acquired immunity without vaccination but having recovered from a known previous infection prior to reinfection; **HybrI:** Hybrid immunity from both vaccination and a known previous infection prior to reinfection.

During Omicron, the decrease in the number of infections for individuals with vaccine-induced immunity was not as pronounced as during Delta (52.8%), but nonetheless followed a similar pattern for infection-induced (87.3%) and hybrid immunity (93.8%). Similarly, the number of hospitalizations was reduced by 79.7%, 94.9%, and 98.0% for vaccine-induced, infection-induced, and hybrid immunity, respectively. In line with the data collected during the wave dominated by the Delta VOC, a similar pattern of reduction was observed for ICU admissions and deaths during Omicron, where hybrid immunity appeared to provide a superior protection against severe disease over infection-induced immunity, which were both superior to vaccine-induced immunity (Figure 2, Supplementary Table 4).

### Increased protection against severe disease from infection-induced and hybrid immunity may be the result of a broader cross-reactive immunity.

**Figure 3.**
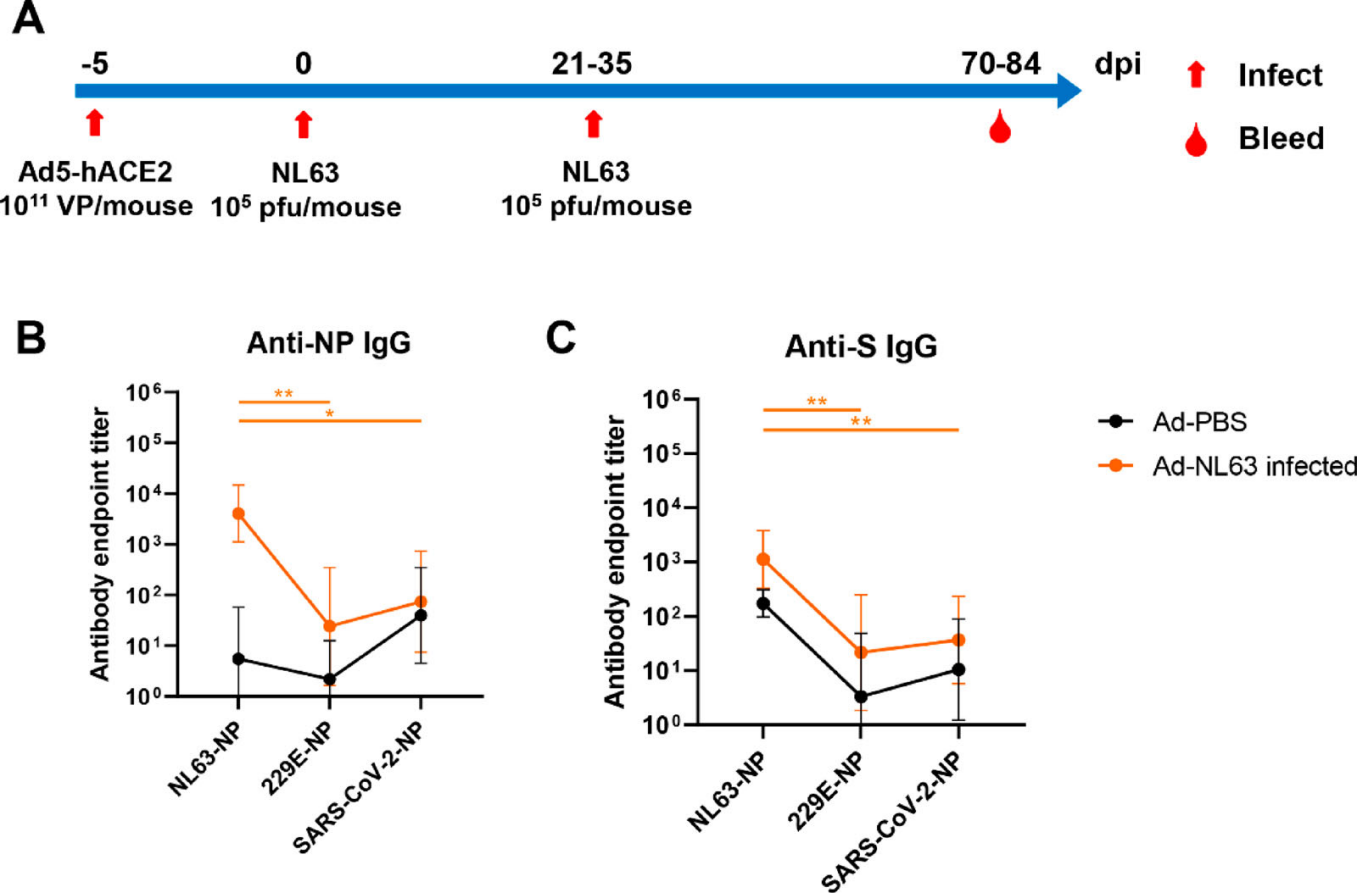
NL63 infected mice produced cross-reactive IgG against HCoV-229E and SARS-CoV-2 antigens. (**A)** Overview of Ad5-hACE2 transduction and NL63 infection of BALB/c mice. (**B, C)** The ELISA plates were coated with N or S. The sera were collected from the Ad-PBS group (*n* = 5) and Ad-NL63 infected groups (*n* = 6). HRP-conjugated goat anti-mouse IgG Ab was used as secondary antibody. The absorbance was read at 450 nm after terminating reaction. Statistical significance was determined by the two-tailed t-test (*p < 0.05, **p < 0.01).

We hypothesized that the apparent increased protection observed in the context of infection-induced and hybrid immunities could be the result of the broader immune responses provided through exposure to numerous viral antigens during infection. As such, we evaluated cross-reactive immunity in an animal model of infection. BALB/c mice transduced with human ACE2 were infected twice with HCoV-NL63, 21 or 35 days apart, and the antisera of infected animals was collected on day 70 or 84 post-infection (Figure 3A). The samples were analysed for cross-reactive IgG to other human coronaviruses, namely HCoV-229E and the prototype SARS-CoV-2, initially reported in Wuhan. Here, we found that the antisera of animals infected with HCoV-NL63 was capable of weakly cross-reacting against the N (Figure 3B) and S proteins (Figure 3C) of both HCoV-229E and SARS-CoV-2, which may be due to the conservation of the N protein and S2 subdomain of the spike protein.

Similarly, we have been investigating the presence of cross-reactive immune responses in pre-pandemic human sera from various countries across multiple continents. Consistent with the animal data presented above, we observed in Brazil, Denmark, Gabon, and Turkey a trend for increased responses towards the nucleocapsid of SARS-CoV-2 compared to the spike protein, which was significant only for Brazil (Table 1).

**Table 1.** Cross-reactivity of pre-COVID-19 sera to the spike (S) and nucleocapsid (N) of SARS-CoV-2.

Country	Cross-reactivity vs S (%)	Cross-reactivity vs N (%)
Brazil*	0/108 (0.0)	4/108 (3.7)
Canada^ns^	1/41 (2.4)	1/41 (2.4)
Denmark^ns^	1/117 (0.9)	3/117 (2.6)
Gabon^ns^	12/116 (10.3)	20/116 (17.2)
Senegal***	20/144 (13.9)	4/144 (2.8)
Turkey^ns^	0/22 (0.0)	1/22 (4.6)

Note: Statistical significance was determined by Chi-squared test (^ns^not significant).

*p < 0.05; ***p < 0.001.

## Discussion

A review of seven clinical and observational studies assessing the protection from infection-induced versus vaccine-induced immunity, as well as the added benefit of hybrid immunity, concluded the net benefit to the vaccination of individuals who had recovered from COVID-19 was merely marginal, and that infection-acquired immunity was, at the minimum, equivalent to that of a fully-vaccinated individual [13]. During the Alpha and Beta variants, the efficacy of natural infection at preventing reinfection was estimated at about 98 and 92%, respectively [14]. The benefits of vaccinating previously infected individuals regarding risk of reinfection, whether homologous or heterologous, remain a source of debate, and the notion of emergency regarding vaccination of these individuals may not be warranted. Indeed, recent evidence shows natural immunity in unvaccinated individuals can be detected up to 20 months following COVID-19 infection [15], which is consistent with observations for SARS-CoV and MERS-CoV, as discussed above. Importantly, a nationwide study conducted in Sweden reached similar conclusions, in which individuals previously infected by SARS-CoV-2 presented a low risk of reinfection and hospitalizations for up to 20 months [16]. Again, these data are supported by a recent analysis during the Delta wave showing only a marginal increase in cases among individuals previously infected regardless of their vaccination status. In contrast, case incidence for individuals infected for the first time increased significantly, suggesting infection-associated immunity may help protect against reinfection [9].

From an immunological standpoint, this is unsurprising. Acquired infection provides a much broader reactivity than spike-specific vaccines, as several other antigens, such as the N, also are presented to the immune system and can be targeted following reinfection. Additionally, structural and enzymatic proteins are more genetically stable than the S protein [17], and thus contain highly-conserved regions among all known and, likely, future SARS-related viruses recognizable by the immune system. For example, studies have described the prevalence of SARS-CoV-2 cross-reactive T cells in naïve individuals [18,19], and a recent evaluation of COVID-19 household contacts even highlighted the role of pre-existing, non-spike cross-reactive T cells in protection [20]. Similarly, our group recently showed the presence of cross-reactive antibodies to the N of SARS-CoV-2 in pre-pandemic serum samples from Africa, a continent comparatively spared from COVID-19 despite its difficult access to advanced health care including vaccine doses [21]. Here, we have updated the data with additional samples from Turkey, and responses towards the N were trending upward compared to responses against the S protein of SARS-CoV-2. In addition, the findings from mice in our study support the potential for protection of pre-existing cross-immune responses against N and S. Lastly, immunization of rats with N was shown to trigger a pulmonary immune response through lymphocyte and macrophage infiltration, which may prove advantageous towards protection [22]. These findings support the inclusion of other antigens, such as N, in future vaccine designs toward creating broader immunity.

The conserved protection from multiple viral antigens against variants and future strains is an area of ongoing research. As mentioned above, cross-clade pan-sarbecovirus Nabs could neutralize several SARS-CoV-2 VOCs as well as other coronaviruses. Still, the cross-protection appears to be variant dependent. For example, the effects of boosting on B and T cell-mediated immunity following infection by Omicron was shown to be dependent on the previous variant of infection. Individuals previously infected with either Wuhan Hu-1, Alpha, or Omicron exhibited abrogated, reduced, or cross-reactive responses, respectively [23]. This VOC variability translates into variability of vaccine cross-protection as well. The odds of becoming infected for those vaccinated compared to unvaccinated were much lower during Delta than for Omicron, likely because of reduced vaccine efficacy given antigenic diversity, as the vaccines were based on the initial Wuhan strain, and the later Omicron strain evolved more dissimilar features. Furthermore, the degree of protection conferred from Omicron infection on upcoming variants is being evaluated for its immune evasiveness resulting from novel mutations within S [24], with results underscoring the need for better cross-reactive vaccines based on multiple viral antigens to combat the latest VOC among such rapidly evolving pathogens beyond short-term protection.

In contrast to infection-induced immunity, the importance and effectiveness of a third dose of mRNA vaccine regarding the prevention of moderately severe and severe COVID-19 has been thoroughly evaluated and supported. However, the efforts of pharmaceutical companies in staying up to date with emerging VOCs may be misplaced, as variant-specific booster shots may not provide significant protection to current and future evolving SARS-CoV-2 viruses. Most studies agree that a single dose of vaccine in previously infected individuals is sufficient to achieve the same level of immunity as two doses administered to naïve individuals [25–27]. Indeed, nonhuman primate (NHP) studies have failed to demonstrate an increased efficacy for Beta- and Omicron-specific booster doses, when compared to an original Wuhan-Hu-1-matched boost [28,29]. Each boost was shown to expand B cells that were cross-reactive to both Omicron and previous strains and resulted in a similar level of protection in an NHP challenge model with Omicron. Similarly, the recent rollout of bivalent booster doses were shown in clinical trials to increase levels of Nabs compared to the previous generation of vaccines [30], but mathematical models failed to predict significant improvements on protection over previous generations of vaccines [31]. Unless major antigenic changes occur, current vaccines appear sufficient to prevent severe disease associated with COVID-19 [32]. As such, current immune responses, either stimulated by vaccination and/or infection have been shown capable of cross-reacting against evolving variants. Understanding how long these immune responses last will be an important factor in prioritizing vaccine allocation among individuals with respect to previous infection status. Toward this end, risk-benefit analyses become crucial. For example, autoantibodies following COVID-19 infection are now gaining more traction for potentially explaining signs and symptoms associated with long-COVID-19 [33–36], which has been described previously for survivors of other pathogens such as Ebola, Zika, and Dengue viruses [37–39]. Accordingly, the effects of repeated booster doses in individuals previously infected by SARS-CoV-2 deserves further evaluation regarding patient health and the potential of exacerbating autoimmune conditions.

Overall, the goal of this analysis was to gain an understanding of the interactions between the various types of immunity to inform the best public health policies to protect individuals and populations against COVID-19. Still, our study is not without limitations. First, individuals who experienced a prior infection (infection-induced and hybrid immunity), may have had other prior infections from the same or other COVID-19 variants. Similarly, the immune responses of individuals were not evaluated prior to classification, so individuals with a reported absence of prior immunity may have experienced an asymptomatic infection that could have led to the development of pre-existing immunity. Secondly, the data we have collected does not inform on the long-term impact of the various types of immunity on incidence and severity of reinfection. Thirdly, the use of aggregated data did not allow for controlling variables such as age, sex, and comorbidities that were demonstrated to impact disease progression following infection. Finally, the total number of individuals in each cohort is relatively small compared to COVID-19 onset, especially for medical parameters such as intensive care unit admissions and deaths. Even so, the UTMB data set is robust due to policy and procedure consistency, as all patients were treated under the UTMB system, providing uniformity in criteria for hospitalization, admission to the ICU, and the determination of COVID-19 as a primary diagnosis. Furthermore, this uniformity within the UTMB study environment solidifies the non-inferiority of natural-immunity protection compared to vaccine alone, a protection closer to hybrid immunity. This is a recurring focus with other pathogens as well, and thus has broad implications toward developing evidence-based policy decisions and consequent private-sector mandates.

In conclusion, this study does not advocate for individuals to purposefully seek natural exposure to such pathogens, even after immunization, and we explicitly caution that such exposure has many unpredictable associated health risks, resulting in more hospitalizations, as supported by this study, in addition to a greater risk of death [40]. Vaccination against COVID-19 has been demonstrated by many to be a formidable tool at an individual health-level, with associated side effects and risks significantly lower than for infection. Even so, this study supports the concept that previously-infected individuals derive limited benefits from the administration of vaccine doses, including boosters. Such acknowledgment should inform population-level policies to account for relevant aspects of personalized medicine unconflated with risky behaviour. Infection-induced immunity can guide public health responses toward alleviating costs and operational difficulties of a blanket vaccination policy in response to future pandemics, particularly during the initial emergency response when vaccines are non-existent or scarce and in demand, ensuring we protect the most vulnerable.

## Supplementary Material

Supplementary_Tables.docx

## Data Availability

The data files used in the current study can be made available upon reasonable request to the corresponding author. It should be noted that the human data will be made available with consideration of a regulated process that oversees human data management at the University of Texas Medical Branch.
