# Supplementary material for: SARS-CoV-2 infection-induced immunity reduces rates of reinfection and hospitalization caused by the Delta or Omicron variants
Source: Emerg Microbes Infect. 2023 Mar 1;12(1):e2169198. doi: 10.1080/22221751.2023.2169198 (PMC9980403; doi:10.1080/22221751.2023.2169198)
Supplement: Supplementary_Tables.docx [file TEMI_A_2169198_SM0065.docx]

# SUPPLEMENTARY TABLES

## Supplementary Table 1.

| **SUPPLEMENTARY TABLE 1. Cohort size of laboratory-confirmed COVID-19 cases in Galveston (N=22,407) and healthcare-associated admissions during the wave caused by the Delta variant - July 01 – October 31, 2020** | | | | | |
| --- | --- | --- | --- | --- | --- |
|  |  |  |  |  |  |
| **Vaccination and diagnosis status^a^** | **No. of positive COVID-19 cases (%)** | **No. of patients visiting ED^b^ (Cumulative incidence)^c^** | **No. of hospitalizations^d^ (Cumulative incidence)^c^** | **No. of ICU admissions^e^ (Cumulative incidence)^c^** | **No. of COVID-19-associated inpatient deaths (Cumulative incidence)^f^** |
|  |  |  |  |  |  |
| ***Unvaccinated*** | ***19,864*** | ***2,160*** | ***886*** | ***161*** | ***127*** |
| NoI | 19,222 (85.8) | 2,121 (11,034) | 879 (4,573) | 161 (838) | 125 (14,221) |
| InfI | 642 (2.9) | 39 (6,075) | 7 (1,090) | 0 (0) | 2 (28,571) |
| ***Vaccinated*** | ***2,543*** | ***135*** | ***52*** | ***12*** | ***8*** |
| VaxI | 2,386 (10.7) | 127 (5,323) | 51 (2,137) | 12 (503) | 8 (15,686) |
| HybrI | 157 (0.7) | 8 (5,095.5) | 1 (637) | 0 (0) | 0 (0) |
| **Total** | 22,407 | 2,295 | 938 | 173 | 135 |
| ^a^The vaccination status is defined by individuals who have received 1 dose of the Jansen, or 2 doses of the Spikevax (Moderna) or the Comirnaty (Pfizer-BioNTech) COVID-19 vaccines. | | | | | |
| ^b^ED patients were discharged home after at most 1 day of observation. If admitted to the hospital, they were included under hospitalizations only. | | | | | |
| ^c^Cumulative cases per 100,000 cases. Incidence is calculated over the dataset period. | | | | | |
| ^d^Hospitalization refers to admission with COVID as primary diagnosis. | | | | | |
| ^e^ICU patients reported are a subset of the patients under hospitalization. | | | | | |
| ^f^The cumulative incidence of inpatient deaths is calculated per 100,000 hospitalizations. | | | | | |

## Supplementary Table 2.

| **SUPPLEMENTARY TABLE 2. Cohort size of laboratory-confirmed COVID-19 cases in Galveston (N=29,070) and healthcare-associated admissions during the wave caused by the Omicron variant - December 10, 2021– February 28, 2022** | | | | | |
| --- | --- | --- | --- | --- | --- |
|  |  |  |  |  |  |
| **Vaccination and diagnosis status^a^** | **No. of positive COVID-19 cases (%)** | **No. of patients visiting ED^b^ (Cumulative incidence)^c^** | **No. of hospitalizations^d^ (Cumulative incidence)^c^** | **No. of ICU admissions^e^ (Cumulative incidence)^c^** | **No. of COVID-19-associated inpatient deaths (Cumulative incidence)^f^** |
|  |  |  |  |  |  |
| ***Unvaccinated*** | ***19,721*** | ***1,544*** | ***372*** | ***63*** | ***37*** |
| NoI | 17,499 (60.2) | 1,414 (8,080) | 354 (2,023) | 61 (349) | 36 (10,170) |
| InfI | 2,222 (7.6) | 130 (5,851) | 18 (810) | 2 (90) | 1 (5,556) |
| ***Vaccinated*** | ***9,349*** | ***367*** | ***79*** | ***6*** | ***5*** |
| VaxI | 8,261 (28.4) | 325 (3,934) | 72 (872) | 6 (73) | 5 (6,944) |
| HybrI | 1,088 (3.7) | 42 (3,860) | 7 (643) | 0 (0) | 0 (0) |
| **Total** | 29,070 | 1,911 | 451 | 69 | 42 |
| ^a^The vaccination status is defined by individuals who have received 1 dose of the Jansen, or 2 doses of the Spikevax (Moderna) or the Comirnaty (Pfizer-BioNTech) COVID-19 vaccines. | | | | | |
| ^b^ED patients were discharged home after at most 1 day of observation. If admitted to the hospital, they were included under hospitalizations only. | | | | | |
| ^c^Cumulative cases per 100,000 cases. Incidence is calculated over the dataset period. | | | | | |
| ^d^Hospitalization refers to admission with COVID as primary diagnosis. | | | | | |
| ^e^ICU patients reported are a subset of the patients under hospitalization. | | | | | |
| ^f^The cumulative incidence of inpatient deaths is calculated per 100,000 hospitalizations. | | | | | |

## Supplementary Table 3.

| **SUPPLEMENTARY TABLE 3. Statistical significance comparisons between the various immunity types, and for each medical parameters assessed, during the wave caused by the Delta variant - July 01 – October 31, 2020** | | | | |
| --- | --- | --- | --- | --- |
| N-Delta | NoI | VaxI | InfI | HybrI |
| NoI |  | **** | **** | **** |
| VaxI | **** |  | **** | **** |
| InfI | **** | **** |  | **** |
| HybrI | **** | **** | **** |  |
| H-Delta | NoI | VaxI | InfI | HybrI |
| NoI |  | **** | **** | **** |
| VaxI | **** |  | **** | **** |
| InfI | **** | **** |  | * |
| HybrI | **** | **** | * |  |
| ICU-Delta | NoI | VaxI | InfI | HybrI |
| NoI |  | **** | **** | **** |
| VaxI | **** |  | *** | *** |
| InfI | **** | *** |  | NS |
| HybrI | **** | *** | NS |  |
| D-Delta | NoI | VaxI | InfI | HybrI |
| NoI |  | **** | **** | **** |
| VaxI | **** |  | NS | ** |
| InfI | **** | NS |  | NS |
| HybrI | **** | ** | NS |  |

**N:** Positive COVID-19 cases; **H:** Hospitalizations; **ICU:** Intensive care unit visits; **D:** Deaths; **NoI**: No known induced immunity from reportedly naïve patients who have not been vaccinated and have not experienced a COVID-19 infection prior to the current infection; **VaxI:** Vaccine-induced immunity prior to first infection; **InfI:** Infection-acquired immunity without vaccination but having recovered from a known previous infection prior to reinfection; **HybrI:** Hybrid immunity from both vaccination and a known previous infection prior to reinfection. Statistical significance was determined by Chi-squared tests (NS: not significant, *p≤0.05, **p≤0.01 ***p≤0.001, ****p≤0.0001).

## Supplementary Table 4.

| **SUPPLEMENTARY TABLE 4. Statistical significance comparisons between the various immunity types, and for each medical parameters assessed, during the wave caused by the Omicron variant - December 10, 2021– February 28, 2022** | | | | |
| --- | --- | --- | --- | --- |
| N-Omicron | NoI | VaxI | InfI | HybrI |
| NoI |  | **** | **** | **** |
| VaxI | **** |  | **** | **** |
| InfI | **** | **** |  | **** |
| HybrI | **** | **** | **** |  |
| H-Omicron | NoI | VaxI | InfI | HybrI |
| NoI |  | **** | **** | **** |
| VaxI | **** |  | **** | **** |
| InfI | **** | **** |  | * |
| HybrI | **** | **** | * |  |
| ICU-Omicron | NoI | VaxI | InfI | HybrI |
| NoI |  | **** | **** | **** |
| VaxI | **** |  | *** | * |
| InfI | **** | *** |  | NS |
| HybrI | **** | * | NS |  |
| D-Omicron | NoI | VaxI | InfI | HybrI |
| NoI |  | **** | **** | **** |
| VaxI | **** |  | NS | * |
| InfI | **** | NS |  | NS |
| HybrI | **** | * | NS |  |

**N:** Positive COVID-19 cases; **H:** Hospitalizations; **ICU:** Intensive care unit visits; **D:** Deaths; **NoI**: No known induced immunity from reportedly naïve patients who have not been vaccinated and have not experienced a COVID-19 infection prior to the current infection; **VaxI:** Vaccine-induced immunity prior to first infection; **InfI:** Infection-acquired immunity without vaccination but having recovered from a known previous infection prior to reinfection; **HybrI:** Hybrid immunity from both vaccination and a known previous infection prior to reinfection. Statistical significance was determined by Chi-squared tests (NS: not significant, *p≤0.05, **p≤0.01 ***p≤0.001, ****p≤0.0001).
